# “Evaluation of ROS1 expression and rearrangements in a large cohort of early-stage lung cancer”

**DOI:** 10.1186/s13000-023-01357-1

**Published:** 2023-05-27

**Authors:** Anne Pernille Harlem Dyrbekk, Abdirashid Ali Warsame, Pål Suhrke, Marianne Odnakk Ludahl, Joakim Oliu Moe, Inger Johanne Zwicky Eide, Marius Lund-Iversen, Odd Terje Brustugun

**Affiliations:** 1 University of Oslo, NO-0316 Oslo, Norway; 2 Department of Pathology, Vestfold Hospital Trust, NO-3103 Tonsberg, Norway; 3 Department of Cancer Genetics, Institute for Cancer Research, The Norwegian Radium Hospital, NO-0310 Oslo, Norway; 4 Department of Pathology, Oslo University Hospital, The Norwegian Radium Hospital, NO-0310 Oslo, Norway; 5 Department of Microbiology/ Division for Genetechnology, Vestfold Hospital Trust, NO-3103 Tonsberg, Norway; 6 Department of Internal Medicine, Vestfold Hospital Trust, NO-3103 Tonsberg, Norway; 7 Department of Oncology, Vestre Viken Hospital Trust, NO-3004 Drammen, Norway

**Keywords:** Lung cancer, ROS1, Targeted therapy, NGS, Immunohistochemistry

## Abstract

**Background:**

ROS1 fusion is an infrequent, but attractive target for therapy in patients with metastatic non- small-cell lung cancer. In studies on mainly late-stage disease, the prevalence of ROS1 fusions is about 1–3%. In early-stage lung cancer ROS1 might also provide a fruitful target for neoadjuvant or adjuvant therapy. In the present study, we investigated the prevalence of ROS1 fusion in a Norwegian cohort of early-stage lung cancer. We also explored whether positive ROS1 immunohistochemical (IHC) stain was associated with certain mutations, clinical characteristics and outcomes.

**Methods:**

The study was performed using biobank material from 921 lung cancer patients including 542 patients with adenocarcinoma surgically resected during 2006–2018. Initially, we screened the samples with two different IHC clones (D4D6 and SP384) targeting ROS1. All samples that showed more than weak or focal staining, as well as a subgroup of negative samples, were analyzed with ROS1 fluorescence in situ hybridization (FISH) and next-generation sequencing (NGS) with a comprehensive NGS DNA and RNA panel. Positive ROS1-fusion was defined as those samples positive in at least two of the three methods (IHC, FISH, NGS).

**Results:**

Fifty cases were IHC positive. Of these, three samples were both NGS and FISH-positive and considered positive for ROS1 fusion. Two more samples were FISH positive only, and whilst IHC and NGS were negative. These were also negative with Reverse Transcription quantitative real time Polymerase Chain Reaction (RT-qPCR). The prevalence of ROS1 fusion in adenocarcinomas was 0.6%. All cases with ROS1 fusion had TP53 mutations. IHC-positivity was associated with adenocarcinoma. Among SP384-IHC positive cases we also found an association with never smoking status. There was no association between positive IHC and overall survival, time to relapse, age, stage, sex or pack-year of smoking.

**Conclusions:**

ROS1 seems to be less frequent in early-stage disease than in advanced stages. IHC is a sensitive, but less specific method and the results need to be confirmed with another method like FISH or NGS.

**Supplementary Information:**

The online version contains supplementary material available at 10.1186/s13000-023-01357-1.

## Introduction

Targeted therapy has been a game-changer in the treatment of metastatic lung cancer, providing effective treatment opportunities and improving overall survival in late-stage disease [1, 2]. However, for early-stage disease, the treatment opportunities have developed more slowly. There is now a focus on the potential for targeted therapy in early-stage disease, and more research is needed on the prevalence and characteristics of relevant targets in this setting.

The ROS1 fusion protein is an attractive therapeutic target for patients with metastatic non-small cell lung cancer (NSCLC). Several therapies are available and recommended by the European Society for Medical Oncology (ESMO) [3] and National Comprehensive Cancer Network (NCCN) [4]. In studies done mostly on advanced stage disease, the prevalence of ROS1 rearrangements in NSCLC is about 1–3% [5–8]. But there is now a growing interest for the prevalence of ROS1 fusions in early-stage lung cancer. For EGFR mutated resected early-stage NSCLC, adjuvant therapy with tyrosine kinase inhibitor have showed promising results [9]. And perioperative targeted therapy can also be feasible for other oncogene addicted NSCLC. Recently, case reports have showed effect of crizotinib as neoadjuvant or adjuvant therapy in a ROS1 translocated setting [10, 11], and there is also ongoing trials investigating this further [12]. This implies that ROS1 fusions in early stage lung cancer can be a target for neoadjuvant or adjuvant therapy in the future.

Two meta-analyses indicate that ROS1 fusion is more common in late-stage disease [6, 8]. It is also shown that the prevalence is higher in women, never-smokers, adenocarcinomas and in patients of Asian ethnicity. Two previous studies on early-stage lung cancer found a prevalence of ROS1 fusion of 0.4–1.2% in adenocarcinomas [13, 14]. These studies were based on one immunohistochemical (IHC) clone and fluorescence in situ hybridization (FISH).

The validity of fusion detection methods, i.e. detection of *clinically* relevant fusions, remains challenging. Until recently, ROS1 FISH analysis was regarded as a gold standard. However, new methods like next-generation sequencing (NGS) have revealed conflicting results [15]. An emerging strategy in fusion detection diagnostics is the reliance on a combination of several methods, including IHC, FISH, NGS and Reverse Transcription quantitative real time Polymerase Chain Reaction (RT-qPCR). NGS is now more widely available and ESMO recommends the use of NGS in patients with non-squamous NSCLC [16]. For detection of ROS1 fusion, RNA-NGS is preferred over DNA-NGS [15, 17, 18].

IHC-based methods are generally feasible, cost-effective and widely available in pathology labs. These methods are based on immunologic principles, where labeled antibodies bind to specific antigens such as cell proteins. When a part of the ROS1 gene is fused with another gene and the kinase domain is included in that fusion, the subsequent gene expression can lead to an increased ROS1 expression. IHC can therefore detect increased protein expression, but it cannot distinguish whether this is the normal/wild type ROS1 protein or a chimeric protein as a result of gene fusion [19]. For ROS1 IHC there is still no consensus on cut-off levels, though several studies have focused on different IHC clones for ROS1-detection [14, 15, 20–23]. Only a few studies have used NGS as part of the test algorithm [15, 20, 22]. To our knowledge, there are no published studies based on a diagnostic algorithm combining different IHC-clones, FISH and comprehensive NGS-panels.

This retrospective study aimed to determine the prevalence of ROS1 fusion in a cohort of Norwegian, early-stage resectable lung cancer. We used ROS1 IHC screening and performed FISH and NGS on IHC positive samples, and described relevant challenges in the interpretation of the test results from all three methods. In cases with positive FISH and negative IHC/NGS cases, we have also used RT-qPCR. In addition, we explored associations between ROS1 fusion or IHC positivity, and clinical, histopathological and comprehensive genetic characteristics.

## Methods

### Patients

We used biobank specimens from a cohort of surgically resected lung cancer patients at Oslo University Hospital. This biobank has a connected database with histopathological-, biological- and clinical information. The surgery and sampling was done during the period 2006–2018, with a median follow-up exceeding 5 years. Mortality data were imported from the Norwegian Population Registry, which is updated monthly. Two patients were lost to follow up (emigration), and was considered negligible in the analysis. We also excluded patients with carcinoid tumour and thymoma. Written informed consent was obtained from all patients and the project was approved by the regional ethics committee (1904/2009).

The staging has been done according to the latest TNM classifications of malignant tumours at the time of surgery, but for the FISH positive cases we restaged the samples to the current edition [24]. For adenocarcinomas with ROS1 fusion, we have also used the proposed new grading system for invasive pulmonary adenocarcinoma [25].

### Specimen characteristics

In this study we used full size slides from formalin-fixed paraffin embedded (FFPE) blocks, tissue micro arrays (TMA), and DNA and RNA extracted from fresh frozen material or FFPE blocks.

The full size slides were taken from FFPE blocks from resections in the diagnostic biobank at Oslo University Hospital. These blocks were sliced at the Department of pathology at Vestfold hospital trust.

Tissue microarray blocks were made from specimens from all the resections, each block consisting of specimens from 25–30 patients. All specimens were represented in triplicates, each core being one mm in diameter, and all tissue cylinders were harvested from the original FFPE blocks after careful selection by a trained pathologist. The TMA blocks were made and sliced at the Department of pathology at Oslo University Hospital. The majority of the TMA blocks have been used in previous projects [26–28].

DNA and RNA were extracted from fresh-frozen tissue obtained at surgery. In the few cases of an insufficient number of tumour cells in the frozen tissue samples, the extraction was done from FFPE-blocks.

Positive external control in ROS1-IHC can be difficult to find [29], because of the lack of reliable naturally ROS1 positivity in normal tissue. We used known positive FFPE tissue from a patient with a CD74-ROS1 fusion (confirmed with FISH and NGS) as a positive control. This tumour tissue was also strong and diffuse positive with the two different ROS1 IHC clones (D4D6 and SP384).

### Assay methods. Immunohistochemistry

We performed ROS1 IHC analyses by use of two different ROS1-directed antibody clones: ROS1 (D4D6) Rabbit mAb (Cell Signaling, 3287) and the VENTANA ROS1 (SP384) Rabbit Monoclonal Primary Antibody (Roche Diagnostics, 790–6087). The slides were stained at the Department of Pathology at Vestfold Hospital Trust on a VENTANA BenchMark ULTRA system. This is a fully-automated IHC staining platform. For details about the protocols, see Additional file 1: Table S1. The microscope was an Axio Imager.A2 (Zeiss, item no. 490022–0009-000).

We used both a qualitative and a semiquantitative scoring system. All samples were initially grouped into one of seven predefined groups; Negative, weak and focal, weak and diffuse, moderate/strong and focal, moderate/strong and diffuse, ambiguous and too few viable tumour cells (<10 viable tumour cells). Negative samples were defined by lack of tumour cell positivity under 400x (40x objective) magnification. Moderate/strong positivity was defined by dark brown staining with 25 - 200x magnification (2,5-20x objective). Weak positivity was defined by light brown staining, visible at 100-200x (10-20x objective), but requiring of 400x (40x objective) to see clearly. Diffusely positivity was defined by more than 50% of tumour cells with positive staining, including weak positivity, while focal positivity was defined by less than 50% of tumour cells with positive staining. Ambiguous samples were samples that could not be easily classified due to unspecific staining. Within the TMAs, the staining intensity and distribution were scored by assessing the cores from each case as one unit.

All ambiguous samples were reexamined by an additional pathologist and were classified in a consensus meeting. To assess the inter-observer variability and ensure standardized categorization, a random sample of five of the TMA-blocks (114 cases) were examined by two pathologists individually. Any discrepancies in the interpretation were noted and discussed in a consensus meeting in order to optimize reliability.

We also used a combinative semiquantitative scoring system on both the TMA slides and full size slides. In order to compare results, we used the H-score [30] which has been used in several recent papers on ROS1 expression [14, 15, 20, 21, 23]. We used the same formula as Huang et al. 2019 [22]: (1x (percentage of relevant cells with 1+ staining) + (2x (percentage of relevant cells with 2+staining) + 3x (percentage of relevant cells with 3+ staining). Staining intensity was defined as absence of staining (0), weak staining (1+), moderate staining (2+) and strong staining (3+). We used the definition of 0, 1+, 2+ and 3+ from Conde et al. 2019 [15]: Negative staining (0), which was defined as an absence of expression; weak staining (1+), which involved the use of a 40x objective; moderate staining (2+), which required the use of a 10x or 20x objective and strong cytoplasmic staining (3+), which was clearly visible with the use of a 2x or 4x objective. See Additional file 1: Table S2 for details about the scoring system and definitions.

To assess the heterogeneity of ROS1 expression, any IHC-positive sample with more than weak and patchy staining was also reexamined with IHC on full size sections from the original FFPE blocks. Heterogeneity in staining can be due to technical issues like fixation, edge artifacts or biological heterogeneity in expression. Thereafter, the IHC-positive samples were grouped according to the percentage of tumour cells with positive IHC and staining quality (membranous/cytoplasmatic/nuclear, granular, diffuse). In addition, in order to reduce false negative IHC due to heterogenic expression, full size section IHC was also performed on a subgroup of TMA-IHC-negative cases where full size sections had already been prepared for a different study (cases with positive NTRK expression in IHC). Heterogeneity was defined as positive cases with areas of both 0 or 1 +  in addition to 2 + or 3 + [15].

### Assay methods. FISH

We tested IHC-positive cases (see definition of IHC-positive under variables) with FISH on full-sized slides. In addition, a sub-group of IHC-negative cases were also tested. FISH was performed at the Pathology department of Oslo University Hospital (Radiumhospitalet) with a dual break apart probe from Abbott (Vysis ROS1 Break Apart FISH Probe Kit, product number 08N29-021). The preparation was done according to the manufacturer’s recommendations [31]. For the interpretation, we chose areas with the best preserved morphology and with clear signals. In the validation/verification of the test, the laboratory has found that a positive tumour is defined with a cut-off of at least 15% split signals, including isolated orange (5`) and green (3`) signals. Fused signals and break apart, single green /orange signals were counted in at least 50 nuclei and also at least 200 signals.

Break apart split signals are green and orange signals separated by at least 1 signal diameter. An isolated 5’or 3’pattern means that an orange or green signal is present alone or together with fused or break apart signals. A fused signal is either break apart signals separated with less than 1 signal diameter or a completely fused signal that appears yellow.

### Assays methods. NGS

NGS was performed on the same samples as FISH. Isolated DNA and RNA were extracted from fresh-frozen tissue (stored at -80 °C) with AllPrep DNA/RNA/miRNA Universal Kit (Qiagen, 80,224) on the QIAcube (automated, spin-column-based nucleic acid extraction from Qiagen). The concentration was measured with Nanodrop (Thermo Fisher Scientific) and Qubit Fluorometer (Thermo Fisher Scientific). We used Agilent 2100 Bioanalyzer (Agilent Technologies) to analyze the RNA-quality. The tumour percentage was evaluated on frozen sections. If the percentage was below 10%, and there were no relevant findings (EGFR-, KRAS- or BRAF-mutation, or ROS1-, ALK- or RET-fusion), then NGS was repeated on slides from FFPE-blocks from the original resection. In these cases, the extraction was done from FFPE blocks using MagLead12gC (Biosystem, A1120) for DNA and Quick RNA FFPE kit (Zymo Research, R1008) for RNA.

For the sequencing, we used the Oncomine Comprehensive V3-panel (OCAv3)(Thermo Fisher Scientific, A35806). This panel covers 161 genes. The library preparation was done on an Ion Chef instrument (Thermo Fisher Scientific). The sequencing was done at both Oslo University Hospital and Vestfold Hospital Trust from July 2019 – September 2021. In Oslo, the sequencing was done on an Ion Torrent S5, and in Vestfold on an Ion Torrent S5XL. For the bioinformatical analysis, we used the Ion Reporter version 5.10–5.16 with a custom filter (Oncomine Variants, 5% CI CNV ploidy >  = gain of 2 over normal). The sequencing of DNA and RNA based on tissue from FFPE blocks was done in Vestfold. In these analyses, the material was more fragmented, and therefore there was more “noise” in the sequencing results. To improve interpretation, we therefore heightened the quality requirements of the original filter in these cases (coverage > 1000 reads, Phredscore > 20, allele frequency above 5%). The same quality requirements were also used in sequencing performed on fresh material, but in these cases the heightened quality requirements were not included in the filter. See Additional file 1: Table S3 for details about the quality requirements. Fusion transcripts with unknown gene partner, can be reported as a non-targeted fusion by a combination of primers used for different targeted isoforms [32].

### Assays method. RT-qPCR

In cases with positive FISH and negative IHC/NGS, RT-qPCR was performed with Idylla GeneFusion Assay RUO/1.1 (Biocartis AO121/6). Eluat of RNA extracted for NGS was used in the procedure. The most common fusion partners (CD74, SDC4, SLC34A2, EZR, TPM3, GOPC and LRIG3) are included in the kit. In addition the method can detect if there is an expression imbalance between the 5´and 3´end of the ROS1 gene. An expression imbalance indicates that there can be a fusion with a partner other than those detected by the kit [33]. 

### Dependent and independent variables

The main outcome of this study was ROS1 fusion positivity and ROS1 IHC-positivity. Positive ROS1-fusion was defined as samples positive in at least two of the three methods (one of the IHC-clones, FISH, NGS). Positive ROS1 IHC-staining was defined as focal moderate/strong, diffuse weak or diffuse moderate/strong staining, while negative ROS1 IHC-staining was defined as clearly negative or focal and weak staining.

The independent variables included the clinical parameters like smoking status (former, current or never smoker), sex and age (continuous), stage, histopathological diagnosis and NGS-results.

### Study design and statistical analysis methods

This was a retrospective, cohort study based on linked register data and biobank material. We examined the data by use of frequency tables. Associations between positive IHC-staining and epidemiological factors and specific mutations were tested by use of univariate logistic regression with positive IHC-staining as the dependent variable. To test for differences in heterogeneity between the two IHC-clones, we used a test for equality of proportions. We regarded two-sided P values < 0.05 as statistically significant. Differences in overall survival and time to relapse were assessed by use of Kaplan Meier plots and log rank test. STATA Release 16 [34] was used for statistical analysis.

## Results

The biobank contained tissue from 921 resection specimens. The median age was 67.5 years (range 39.2 to 87). The majority (93.1%) were current or former smokers with a median pack-year of 32.9. 83.7% were in stage I and II and 16.1% were in stage III and IV. The three most common histology subtypes were adenocarcinoma (58.8%), squamous cell carcinoma (32.6%) and large cell carcinoma (3.0%). See Table 1.

**Table 1:** Demographic-, clinical and histopathological variables at baseline

	**All frequency,** **n (%)**	**ROS1 IHC D4D6** **Total: 891 Missing:30**		**ROS1 IHC SP384** **Total: 902 Missing 19**	
		**Positive**	**Negative**	**Positive**	**Negative**
**Total**	921 (100%)	28 (3.1%)	863 (96.9%)	40 (4.4%)	862 (95.6%)
**Age, median years (range)**	67.5 (39.2–87.0)	67.8 (51.3–81.6)	67.5 (39.2–87)	68.4 (46.4–82.6)	67.5 (39.2–87)
**Sex**					
Male	474 (51.5%)	13 (46.4%)	447 (51.8%)	19 (47.5%)	444 (51.5%)
Female	447 (48.5%)	15 (53.6%)	416 (48.2%)	21 (52.5%)	418 (48.4%)
**Smoking status**					
Never smoker	64 (6.9%)	3 (10.7%)	61(7.1%)	11 (27.5%)	53 (6.1%)
Current/former smoker	857 (93.1%)	25 (89.3%)	802 (92.9%)	29 (72.5%)	809 (93.9%)
** Median pack-year**	32.9	24	31.5	23.8	31.5
**pStage**					
Ia and b	501 (54.4%)	17 (60.7%)	463 (53.7%)	26 (65%)	462 (53.6%)
IIa and b	270 (29.3%)	8 (28.6%)	256 (29.7%)	8 (20.0%)	257 (29.8%)
III a and b	137 (14.9%)	2 (7.1%)	132 (15.3%)	5 (12.5%)	131 (15.2%)
IV	11 (1.2%)	1 (3.6%)	10 (1.2%)	1 (2.5%)	10 (1.2%)
Unknown stage	2 (0.2%)		2 (0.2%)		2 (0.2%)
**Histology**					
Adenocarcinoma (incl. former bronchioalveolar carc.)	542 (58.8%)	27 (96.4%)	495 (57.4%)	37 (92.5%)	492 (57.1%)
Adenosquamous	16 (1.7%)	0	16 (1.9%)	0	16 (1.9%)
Large cell carcinoma	28 (3.0%)	0	27 (3.1%)	0	27 (3.1%)
Squamous cell carcinoma	300 (32.6%)	0	291 (33.7%)	3 (7.5%)	292 (33.9%)
Other	35 (3.8%)	1 (3.6%)	34 (3.9%)	0	35 (4.1%)

The majority of patients had adenocarcinoma or squamous cell carcinoma and were in stage I and II. Missing: Samples with too few viable tumourcells in the TMAs

### Distribution of tumour markers

**Fig. 1:**
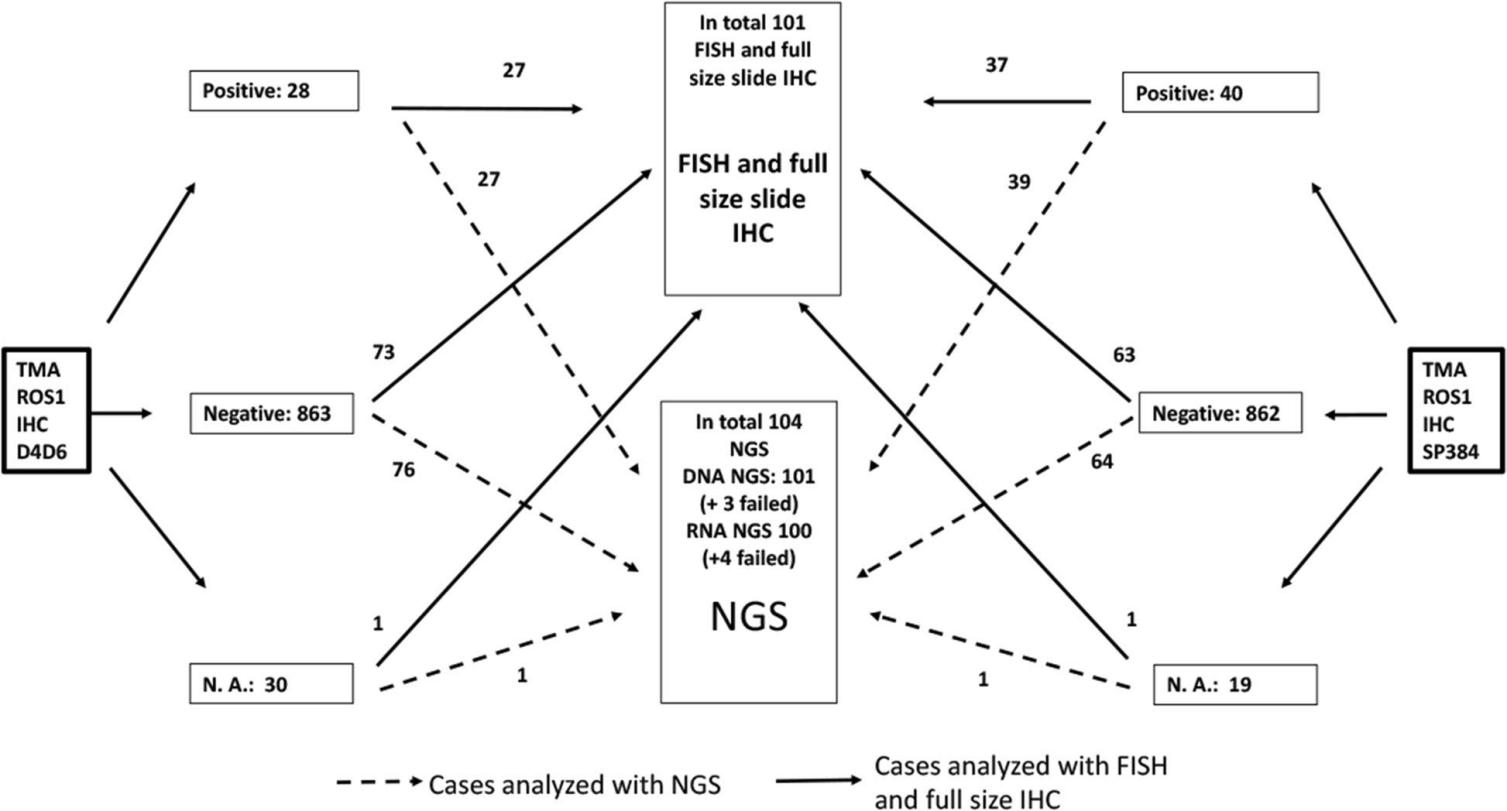
Flowchart. The flowchart shows how many samples that has been tested with FISH and NGS. N.A.: Not applicable because of too few tumourcells

We made 36 TMAs of the samples. Some of the cores in the TMA contained too few tumour cells to interpret the immunohistochemical analyses (32 for D4D6 and 21 for SP384). Twenty eight (D4D6) and forty (SP384) cases showed more than weak and focal staining and were defined as positive IHC. These cases went forward to further testing with FISH, NGS and full size section IHC. In addition, several negative and weak and focal cases were analyzed with this expanded testing (Fig. 1).

In total, 50 of the 921 cases were identified as positive by at least one of the IHC clones. Among these, 18 cases were identified as positive by both clones, whereas 10 of the D4D6-positive cases were negative with SP384 and 22 of the SP384-positive cases were negative with D4D6. SP384 had a higher number of IHC-positive cases than D4D6. In each scoring group except in the group with diffuse, moderate/strong staining, the mean H-score was higher with SP384 (Table 2).

**Table 2:** Distribution of tumour markers

Scoring group	Totally negative	Focal, weak	Focal, moderate/strong	Diffuse, weak	Diffuse, moderate/strong	No viable tumour cells
D4D6 TMA N (%)	837 (90.9)	26 (2.8)	1 (0.1)	19 (2.1)	8 (0.9)	30 (3.3)
Mean/median H-score (range)	0	18.7/20 (5–40)	35/35 (35)	82.6/80 (60–130)	221.3/220 (160–300)	
FISH positiv	2/68	0/5	0/1	0/19	3/7	0/1
NGS	69/837	7/26	1/1	18/19	8/8	1/30
–RNA/ Fusions	ALK: 1 FGFR3: 1 MYB: 1 RET: 1 Failed: 2 Negative: 63	Negative: 6 Failed: 1	Negative: 1	Met exon 14 skipping: 1 Failed: 1 Negative: 16	ROS1: 3 Negative: 5	Negative: 1
SP384 TMA N (%)	827 (89.8)	35 (3.8)	1 (0.1)	24 (2.6)	15 (1.6)	19 (2.1)
Mean/median H-score (range)	0	25.3/30 (5–40)	40/40 (40)	100.4/100 (60–140)	204/200 (120–300)	
FISH positive (Positive/total tested)	2/56	0/7	0/1	0/21	3/15	0/1
NGS	60/827	4/35	1/1	23/24	15/15	1/19
–RNA/Fusions	FGFR3: 1 MYB: 1 Failed: 2 Negative: 56	RET: 1 Negative: 3	Negative: 1	ALK: 1 MET: 1 Failed:1 Negative: 20	ROS1: 3 Failed: 1 Negative: 11	Negative: 1

Negative IHC is defined as totally negative or focal and weak staining. In this table we have separated the negative group in totally negative and focal and weak. For details about the results of the DNA NGS (copy number variation (CNV) and hotspot mutations), see Additional file 1: Table S5

The interobserver agreement was high in determining whether the cases were either negative/focal and weak/not enough tumour cells or strong/diffusely weak, with an observed agreement of 0.99 for D4D6 and 0.97 for SP384. The discrepant cases were mostly due to different interpretation of reactive pneumocytes and macrophages.

We performed 101 FISH analyses, with 27 and 37 of the D4D6 and SP384 positive cases respectively. FISH found five cases of ROS1-fusion. The percentage of positive FISH signals ranged from 16–64%. Surprisingly, two cases that were negative in IHC with both clones showed a split pattern (above 15%) with FISH. These two cases (case number 2 and 3, Table 3), were also completely IHC-negative (H-score 0) on the full slide sections.

**Table 3:** Cases with positive FISH

Case	**FISH** **signals (%)**	**IHC H-score TMA/Fullslide**		IHC heterogeneity	DNA-NGS	RNA-NGS	Histology	pStage	Age/ sex	Smoking status/ Pack-year
		**D4D6**	**SP384**							
1	39	240/300	300/300	No	TP53 AKT2 CNV	ROS1-CD74	Adenocarcinoma (mucinous component)	IIb	75.1/male	Former smoker/12.3
2	28	0/0	0/0	No	Fail	Fail	Squamous carcinoma	IIIa	69.4/male	Former smoker/34.5
3	16	0/0	0/0	No	Fail	Negative	Adenosquamous	IIb	58.1/female	Current smoker/55
4	64	300/300	300/300	No	SETD2, TSC2, TP53	ROS1-CD74	Adenocarcinoma (Solid)	IIIa	61.6/female	Never smoker/0
5	53	200/300	200/300	No	PTCH1, TP53	ROS1-CD74	Adenocarcinom (mucinous component)	IIb	61.7/female	Current smoker/23.5

In the column *FISH signals* we report the sum of the isolated 3`signal, isolated 5`signal and split signals divided on the total number of signals (the equation is described under Methods and in Additional file 1)

One of the cases had a percentage of isolated/split signals of 16% (just above the threshold). This case was also negative with RNA-NGS. The other case had a percentage of isolated/split signals of 28%, which is low, but considered clearly positive. In this last case the NGS failed on FFPE material and fresh frozen tissue was not available. RT-qPCR was negative on these two discrepant cases, and there were no 3´-5´ ROS1 expression imbalance. The cases with positive FISH were in stage IIb-IIIa [24]. The adenocarcinomas are all grad 3/poorly differentiated tumours with predominantly (> 20%) high grade pattern (solid and trabecular/complex glandular patterns) [25].

With NGS we detected three cases with ROS1-fusion. These cases (case number 1, 4 and 5, Table 3) showed a strong, granular, cytoplasmatic staining without membranous attenuation and no heterogeneity with both clones and they were all FISH positive. All three cases had a TP53 mutation, the CD74 –gene was the fusion partner and they were all adenocarcinomas in stage IIb or IIIa. As shown in Fig. 1, 76 of the D4D6 and 64 of the SP384 negative samples were also analyzed with NGS, but we found no ROS1 fusion in this group.

**Fig. 2:**
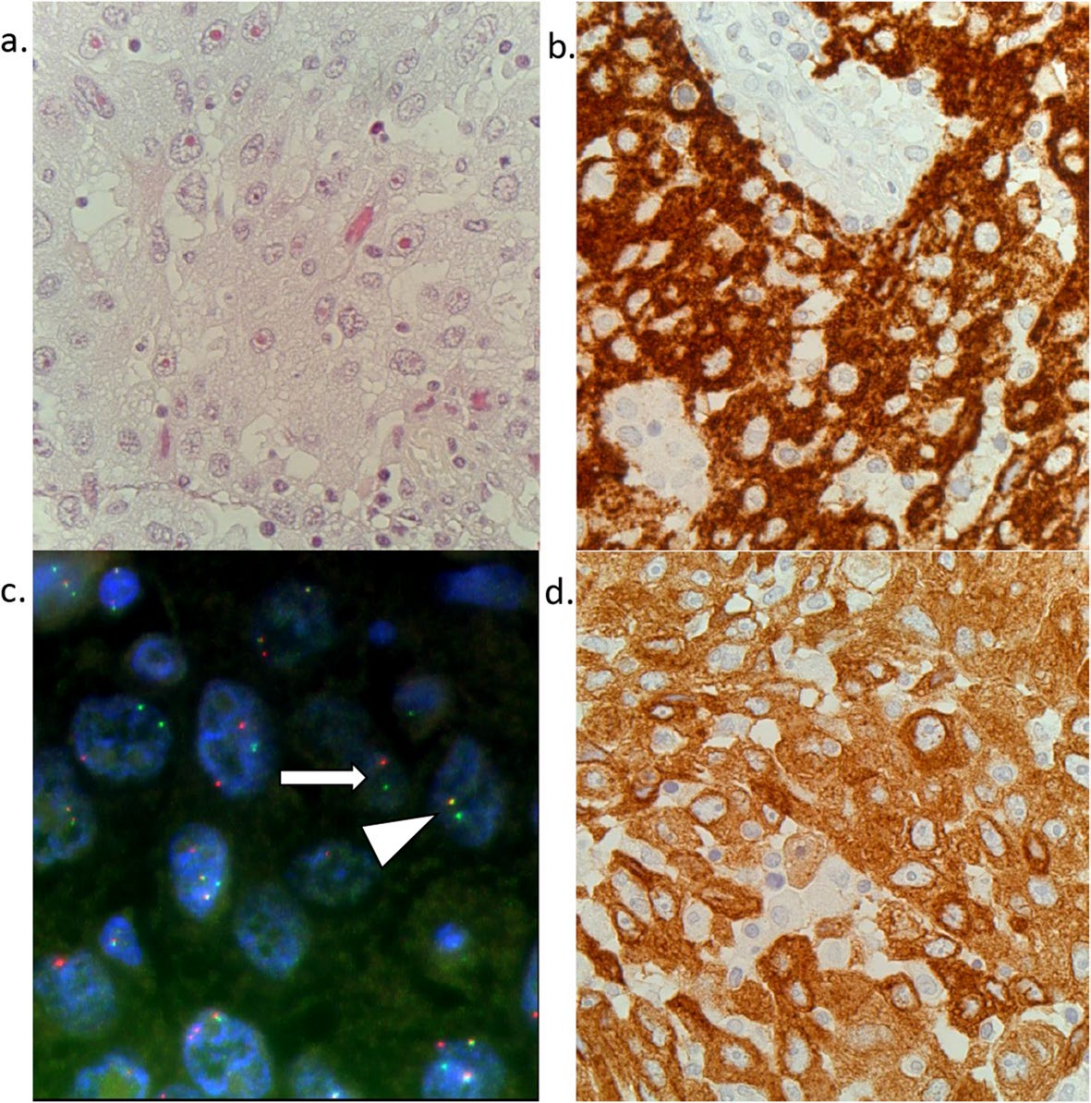
Microscopic pictures. **a** Hematoxylin azophloxine saffron (HAS) staining (400x). Adenocarcinoma with confirmed ROS1 fusion. **b** Same case with strong and diffuse positivity with D4D6 (400x). **c** FISH Arrow: Split signals. Arrowhead: One fused signal and one single green signal. **d** Strong and diffuse positivity with SP384 (400x)

Three cases were positive in at least two of the three methods (IHC, FISH, NGS). These were considered confirmed cases with ROS1-fusions according to our definition of ROS1-fusion positive (samples positive in at least two of the three methods). All of these cases were positive in all three methods. They showed diffuse and strong staining intensity, and all of them were adenocarcinomas (Fig. 2). Thus, the prevalence of ROS1 fusion was 0.6% in adenocarcinomas and 0.3% in the whole cohort.

There was no significant differences in heterogeneity between D4D6 and SP384 (*p* = 0.967). The proportion of positive cases with heterogenic staining was 48.3% and 48.8% for D4D6 and SP384 respectively. None of the three ROS1-fusion confirmed cases showed heterogenic staining.

Eight cases showed strong and diffuse staining with D4D6. Three of five cases where we could not confirm a ROS1 rearrangement showed a partly lepidic or acinar/tubular growth pattern in contrast to the confirmed cases that had solid or trabecular growth patterns. The non-confirmed cases also showed more heterogenic staining on the full size slides and H-score on these slides were from 100–240. We found the same pattern with SP384 where ten of twelve non-confirmed cases showed a lepidic or acinar/tubular growth pattern, and more heterogenic staining on full size slides.

### Expression and ROS1 fusion related to standard prognostic variables, genetics, relapse and overall survival

Positive ROS1 IHC-staining was strongly associated with adenocarcinomas. The estimated ORs for positive IHC-staining was 20.1 (95% CI 2.7–148.4, *p* = 0.003) and 9.3 (95% CI 2.8–30.1, *p* < 0.001) for the D4D6 clone and the SP384 clone respectively. Positive IHC-staining with the SP384 clone was statistically less frequent among former and current smokers than never-smokers (OR 0.2, 95% CI 0.08–0.36, *p* < 0.001). The same association was not found for the D4D6 clone. For both IHC-clones, there were no associations between positive staining and pack-year of smoking, stage, sex or age.

We found TP53 mutation in all three cases with confirmed ROS1 fusions, and they had no other driver mutations like KRAS, BRAF or EGFR. Among the 104 NGS analyzed cases, the two most frequent mutations were TP53 (*n* = 49) and KRAS (*n* = 20). IHC-positivity was associated with KRAS mutation, both in D4D6 (OR 6.5, 95% CI 2.3–18.7, *p* = 0.001) and SP384 (OR 2.9, 95% CI 1.1–8.1, *p* = 0.04), but when we adjusted for adenocarcinoma histology there was no significant association. There were no statistically significant associations between positive IHC and TP53.

There was no statistically significant association between IHC expression and overall survival or time to relapse.

Two of the patients with ROS1 fusion (case number 4 and 5, Table 3) received adjuvant chemotherapy. Patient number 1 relapsed after 3 years, and died of lung cancer one month later. Patient number 5 relapsed after only 3 months, and died of lung cancer shortly after that. While patient number 4 has still not relapsed and is still alive eleven years after surgery.

One of the two patients with positive FISH and negative IHC/RT-qPCR/NGS (case number 2, Table 3) never relapsed and died after 14 years (other cause). Patient number 3 relapsed after almost 14 months and died 6 months later. None of these two were tested for ROS1 fusion.

## Discussion

In this study we aimed to map the prevalence of ROS1 rearrangement in a Norwegian cohort of early-stage resectable lung cancer, and see whether the tumours with ROS1 fusion or ROS1 protein expression were associated with specific epidemiological, histological or genetic characteristics.

The prevalence of ROS1 fusion in resected adenocarcinomas in this cohort was 0.6%. The SP384 was in general more often positive and therefore less specific, but both clones identified the three cases that were positive with both FISH and NGS. Positive staining was strongly associated with adenocarcinomas. Most of the cases with strong and diffuse ROS1 expression where we could not confirm a ROS1 fusion had an acinar or lepidic growth pattern, in contrast to the confirmed cases that showed solid or trabecular growth pattern. In SP384, positive ROS1 IHC-staining was more common among never-smokers than among former- and current smokers, but this association was not found when using the D4D6 clone. There were no associations between IHC based ROS1 expression and other prognostic markers like age, sex, stage or pack-year of smoking.

### Strengths and limitations

This study was based on comprehensive cohort data from a large number of resected lung cancer patients, with the use of multimodal testing by use of two different IHC-clones, FISH, NGS and RT-qPCR. We believe these features are strengths of the study.

There are several limitations in this study, including the use of TMA and IHC as a screening method, the interpretation of IHC and FISH and detection of fusions with novel/uncommon partners. First, we used TMA in the IHC-based ROS1-screening process. TMA is a cost effective method for IHC-based mass screening. However, morphology assessment can be challenging on IHC-slides and even more challenging on TMAs. Morphology assessment is particularly important as macrophages and reactive pneumocytes can stain positive for ROS1. To reduce the risk of false positive staining, we included a slide from the TMA block with principal staining (hematoxylin and eosin stain), so that the morphology could be examined together with the IHC.

Second, since we only had a small area of the tumour in a TMA, tumour heterogeneity could give a wrong impression of the general protein expression in the entire tumour. The degree and direction of such heterogeneity related sampling bias in our data are unknown. However, previous studies have shown substantial correlation between TMA and full size slides [35]. In our study, the correlation between TMA and full size slides was also good (see Additional file 1: S7). Furthermore, we found that the NGS and FISH positive cases had a homogenous staining pattern, and this is consistent with other studies, especially with the SP384 [15, 21].

Third, we performed manual IHC scoring. Several conditions may affect the subjective interpretation of the intensity of staining. As highlighted by Giltnane et al., background staining, stromal staining and the order in which a slide is observed may be of importance [36]. In our experience, the calibration will also differ from day to day. The subjective assessment of IHC-staining represents a risk of misclassification bias. We attempted to reduce the impact of misclassification bias by using a precise definition in addition to having two pathologists independently calibrate the interpretation. One of the pathologists scored all the TMA twice to reduce the impact of day to day differences in calibrations.

In the FISH interpretation, we used the number of split apart signals/isolated signals and not the number of positive cells which has been more commonly used in previous studies [14, 15, 20, 21, 23]. For FFPE slides, tangentially cut nuclei can give both false negative (split signals not presented on the slide) and false positive results (fused signals are separated in the cutting giving an impression of a single signal). In the validation of the method, the laboratory found that this could be compensated for by counting signals instead of positive cells, an approach for which they have long experience. For cytological specimens, the other formula with a score based on the number of positive cells can be used, as whole nuclei can be evaluated.

Finally, as mentioned in the introduction, for sensitive fusion detection RNA-NGS is preferred over DNA-NGS [15, 17, 18]. However, for targeted RNA-panels like OCAv3, the sensitivity is best if the partner gene is known[37]. We can therefore not totally exclude false negative NGS in the cases with uncommon/novel fusion partners. Nevertheless, the OCAv3 can potentially detect novel/uncommon fusion partners by combinations of primers in the panel, and they are then reported as a non-targeted fusions. We did not find any non-targeted fusions. In addition, in cases with positive FISH and negative NGS/IHC, we performed RT-qPCR with the ability to find evidence of uncommon fusion partners with the 3´and 5´ expression imbalance. No cases with expression imbalance were detected.

### Unexpected findings

Two cases were positive with FISH, but negative with IHC and RT-qPCR. One of them was also negative by NGS and the other one failed. It is known that IHC can give false positive results, and that ROS1 IHC always has to be confirmed with a supplementary test such as RT-qPCR, FISH or NGS. Even though FISH is considered to be the gold standard, other studies also found that positive FISH results are not always reproducible [15]. In cases with isolated 3`pattern there is a biological explanation for this, because this may represent a deletion of the segment of the gene containing the binding site for the 5`probe [15, 38, 39]. In cases with positive FISH and negative IHC, the fusion may have been inactivated after posttranslational modification [21] or it might be a non-functioning fusion where the kinase domain is not included in the fusion [29]. Since FISH break apart probes can detect fusions independent of the fusion partner, the discrepant cases might also be a result of false negative RT-qPCR and NGS. As mentioned earlier, novel/uncommon fusion partners can give rise to false negative RT-qPCR and NGS. However, both the Idylla GeneFusion assay (RT-qPCR) and OCAv3 (targeted RNA-NGS) may manage to detect non-targeted fusions were the fusions partner is not included in the kit [32, 33]. Thus, the negative NGS-finding and in addition negative RT-qPCR may imply that there are true false positive results from the FISH-analysis. None of these two patients have been treated with ROS1 inhibitors.

In total, 50 of the 921 cases were positive by IHC, but only three cases could be confirmed with NGS and FISH. Thus, positive IHC staining is much more common than the presumably clinically relevant ROS1 fusions in this study. In addition to the 47 FISH- and NGS negative cases with IHC-positive staining, we noted unspecific staining in macrophages, osteoclasts and reactive pneumocytes. As discussed, there might be several reasons for unspecific staining [19]. The different clones react to different epitopes on the antigen. The antigen, in this case, is the ROS1 protein, either wild type or chimeric/fused. Chromosomal rearrangement leads to activation of the ROS1-gene with sub-sequent overexpression of the chimeric ROS1 protein. Thus, IHC positive cases in wild type tissue/tumour cells may be due to either preanalytical conditions (too long or short fixation, crushed tissue, etc.), cross reactivity to other epitopes, or biological factors (necrosis, phagocytosis of antigens by macrophages, etc.) [19]. In addition, wild type ROS1 protein overexpression could also explain IHC-positivity, and ROS1 seems to be particularly enriched in lung tissue [40]. Lung cancer seems to be even more enriched, perhaps through genetic or epigenetic mechanisms [41]. Contrary to what is seen in ERBB2 amplification and Her-2 overexpression [42], ROS1 amplification does not seem to affect the ROS1 protein expression [43].

Based on our definition of fusion positivity (2 of 3 tests positive), we found no false negative FISH or NGS. False negative FISH might be because of complex fusions where signals appear normal. Biological reasons for false negative FISH are mostly due to intrachromosomal fusions like GOPC, also called FIG [15, 44].

False negative RNA-NGS is mainly seen in specimens with poor RNA quality, and it is important to evaluate the quality metrics in each sample [15]. The reasons for discrepancies in our two cases with FISH positivity are not clear. In both cases, we found a mix of isolated 3’signals and split signals, but in one of the cases, the percentage was just above the threshold.

### Discussion on how the results from the study integrate with the existing evidence

In this Norwegian cohort of early-stage lung cancer, the prevalence of ROS1-fusion in adenocarcinomas was 0.6%. This is consistent with other studies on early-stage disease. In the study by Selinger et al., 0.4% (1/278) of resected stage I-III adenocarcinomas had a ROS1 fusion and Warth et al. (both used ROS1 IHC and FISH) found a prevalence of 1.2% (5/405) in resected adenocarcinomas [13, 14]. In Warth`s study the majority of patients were in stage I-III, but 2.5% were in stage IV. Bergethon et al. (Reverse Transcription PCR /Sanger sequencing and FISH) found a prevalence of 0.6% in stages I and II and 2.8% in stage III and IV [5].

The three cases with confirmed ROS1 fusion had an H-score between 200 and 300 in both clones, and there was no heterogenic staining. Other studies that have used the two different ROS1 clones also found that the clones had good sensitivity, but the specificity was lower depending on the cut-off level [15, 23].

The average H-score is higher for SP384 in both studies, which is consistent with our findings. The two studies have used the same dilution, detection system and staining platform as we have. While Conde et al. also found that the D4D6 showed a more heterogenic staining pattern in ROS1-positive tumours, statistical difference in heterogenic staining was not found in our material. Both Conde et al. and Hofman et al. have reported a moderate or good interobserver agreement and that positive staining can be found in normal lung tissue as well, which is also consistent with our findings [15, 23]. In ROS1 FISH negative cases they also found some degree of staining in 13–32.1% and 3–20.3% cases analysed with SP384 and D4D6, respectively. Although mostly weak or focal, this emphasizes that ROS1 IHC can show of unspecific staining in non-rearranged cases.

We found a strong association between adenocarcinoma histology and IHC positivity, and for SP384 an association with never smoking. There was no association between positive IHC and overall survival, time to relaps, age, sex, stage or pack-year of smoking. Warth et al. also found an association with adenocarcinoma histology. They also found that ROS1 positivity was associated with longer overall survival and female sex [13]. One reason for this discrepancy may be that they defined positive IHC as cases showing any positivity, while we defined positive as more than focal weak staining.

All three cases with ROS1 fusion had a TP53 co-mutation and the CD74-gene as fusion partner. TP53 is known to be the most frequent co-mutation in ROS1 fusion and is associated with a shorter progression free survival with firstline crizotinib therapy [45]. CD74 is the most common ROS1 fusion partner [46].

## Conclusion

The ROS1 prevalence in adenocarcinomas was 0.6% with our algorithm. Both IHC-clones showed strong and homogenous staining with an H-score above 200 in the three cases where both FISH and NGS confirmed the presence of ROS1 fusion. 50 of 921 cases were positive with IHC, but only three of these cases were confirmed with NGS/FISH. The cases with confirmed fusion showed a solid/trabecular growth pattern, in contrast to most of the non-confirmed cases that showed a lepidic or acinar/tubular growth pattern. We also found ROS1-FISH positive cases that could not be confirmed with IHC, NGS or RT-qPCR. This illustrates that we might need dual testing with two different modalities.

Currently, the prevalence of ROS1 fusion in late-stage disease in the Norwegian population is unknown, but as ROS1 testing has become mandatory in adenocarcinomas we will know more about this in the coming years. Relevant tests should be able to identify treatment responsive tumours. FISH ROS1 has been considered to be the “gold standard”, however we need more data from treatment studies to determine which test and algorithm identifies the clinically relevant ROS1 positive tumours. Hopefully, more trials will lead to broader therapy options for both early and late-stage lung cancer .

## Supplementary Information

**Additional file 1: Table S1.** Details on immunohistochemical protocols. **Table S2.** Details about the scoring system and definitions. Equation for a positive/rearranged ROS1 tumour with FISH. **Table S3.** Quality requirements NGS. **Table S4.** Comparing D4D6 and SP384. **Table S5.** Table including DNA NGS with CNV and hotspot mutation. **Table S6.** IHC and stage, sex and smoking. **Table S7.** TMA versus full size slides.

## Data Availability

Some of the data generated or analyzed during this study are included in this published article and its additional information files. Further datasets are not publicly available. However, they can be made available from the corresponding author on reasonable request and after approval by the Regional Ethics Committee.

## Abbreviations

IHC: Immunohistochemistry
FISH: Fluorescence in situ hybridization
NGS: Next-generation sequencing
RT-qPCR: Reverse Transcription quantitative real time Polymerase Chain Reaction
NSCLC: Non-small cell lung cancer
ESMO: European Society for Medical Oncology
NCCN: National Comprehensive Cancer Network
TMA: Tissue microarray
FFPE: Formalin-fixed paraffin embedded
N.A: Not applicable
CNV: Copy number variation
HAS: Hematoxylin azophloxine saffron
OR: Odds ratio
CI: Confidence interval
