# Supplementary material for: “Evaluation of ROS1 expression and rearrangements in a large cohort of early-stage lung cancer”
Source: Diagn Pathol. 2023 May 27;18:70. doi: 10.1186/s13000-023-01357-1 (PMC10224579; doi:10.1186/s13000-023-01357-1)
Supplement: Supplementary file 1 — Additional file 1: Table S1. Details on immunohistochemical protocols. Table S2. Details about the scoring system and definitions. Equation for a positive/rearranged ROS1 tumour with FISH. Table S3. Quality requirements NGS. Table S4. Comparing D4D6 and SP384. Table S5. Table including DNA NGS with CNV and hotspot mutation. Table S6. IHC and stage, sex and smoking. Table S7. TMA versus full size slides. [file 13000_2023_1357_MOESM1_ESM.docx]

Additional file 1.

Table of contents

[Table S1. Details on immunohistochemical protocols 1](#_Toc133739045)

[Table S2. Details about the scoring system and definitions 2](#_Toc133739046)

[Equation for a positive/rearranged ROS1 tumor with FISH 2](#_Toc133739047)

[Table S3. Quality requirements NGS 2](#_Toc133739048)

[Table S4. Comparing D4D6 and SP384 3](#_Toc133739049)

[Table S5. Table including DNA NGS with CNV and hotspot mutations 4](#_Toc133739050)

[Table S6. IHC and stage, sex and smoking 6](#_Toc133739051)

[Table S7. TMA versus whole sections 6](#_Toc133739052)

##

## Table S1. Details on immunohistochemical protocols

Ventana ROS1 SP384 (790-6087)

| Detection system | Antigen Retrieval | Amplifier | Dilution | Incubation | Staining platform | Counter staining |
| --- | --- | --- | --- | --- | --- | --- |
| OptiView DAB IHC Detection Kit (760-700) | CC1 (On board)  Temp: 100 ^o^C  64 min | No amplifier | Ready to use (RTU) | 16 min  36 ^o^C | Ventana  Benchmark Ultra | Instant Hematoxilin Kit. Shandon (Ref 6765015)  1 min. Manually. Dilution (1:2) |

Cell Signaling ROS1 D4D6 (3287s)

| Detection system | Antigen Retrieval | Amplifier | Dilution | Incubation | Staining platform | Counter staining |
| --- | --- | --- | --- | --- | --- | --- |
| OptiView DAB IHC Detection Kit (760-700) | CC1 (On board)  Temp: 99 ^o^C  64 min | OptiView Amplification Kit (760-099)  8+8 min | Ventana Antibody Diluent (251-018)  1:50 | 32 min  36 ^o^C | Ventana  Benchmark Ultra | Instant Hematoxilin Kit. Shandon (Ref 6765015)  1 min. Manually. Dilution (1:2) |

## Table S2. Details about the scoring system and definitions

| Group | Definition |
| --- | --- |
| Negative | No visible staining under 400x magnification. |
| Weak (1+) and focal | Light brown staining, visible at 100-200x (10-20x objective), but requiring of 400x (40x objective) to see clearly in <50% of tumor cells. |
| Weak (1+) and diffuse | Light brown staining, visible at 100-200x (10-20x objective), but requiring of 400x (40x objective) to see clearly in >50% of tumor cells. |
| Moderate/strong (2+ and 3+) and focal | Dark brown staining with 25 - 200x magnification in <50% of tumor cells |
| Moderate/strong (2+ and 3+) and diffuse | Dark brown staining with 25 - 200x magnification in >50% of tumor cells |
| Too few viable tumor cells | <10 viable tumor cells |

## Equation for a positive/rearranged ROS1 tumor with FISH

$$\frac{Isolated 5` signal+isolated 3`signal+break apart split signals}{Isolated 5` signal+isolated 3`signal+break apart split signals+fused signals}x100>15\%$$

## Table S3. Quality requirements NGS

Quality requirements NGS

| **TORRENT SUITE** |  |
| --- | --- |
| % Loading | At least 60% |
| % Polyclonality | 45% or less |
| Low Quality | <20% for DNA, higher for RNA because of degraded material |
| Total Reads | DNA+RNA: 45 000 000-75 000 000 |
| Median reading lenght for DNA | 110-115 bp |
| Medium reading lenght for RNA | 65-110 bp |
|  |  |
| **DNA** |  |
| Mapped Reads | 3 000 000- 6 000 000 |
| % reads on target | >90% |
| Mean depth | >1200 |
| Uniformity | >95%, for degraded material: 85-90% |
| Base coverage depth | >800 bp |
| Number of amplicons | 3781 |
|  |  |
| **RNA** |  |
| Mapped Reads | 500 000-1 500 000 (minimum 40 000) |
|  |  |
| **ION REPORTER** |  |
| **DNA** |  |
| Mutation (QC) | Phred QUAL Score >20  Coverage>1000 |
| CNV | Copy number ≥5 |
|  | MAPD <0,5 |
|  |  |
| **RNA** |  |
| Total Mapped Fusion Panel Reads | >500 000 |
| Expression control counts | Read counts >15 |
| Expression control detected | In total 6 (3 i each pool, should see at least 2-3.) |
| Fusion | Read counts >1000 |
| Skipping and deletions | Read counts >1000 |

## Table S4. Comparing D4D6 and SP384

|  |  | SP384 TMA | | |  |
| --- | --- | --- | --- | --- | --- |
|  |  | Pos | Neg | No viable tumor cells | Total |
| D4D6 TMA | Pos | 18 | 10 | 0 | 28 |
|  | Neg | 22 | 834 | 7 | 863 |
|  | No viable tumor cells | 0 | 18 | 12 | 30 |
|  | Total | 40 | 862 | 19 | 921 |

|  |  | SP384 TMA | | | | | | |
| --- | --- | --- | --- | --- | --- | --- | --- | --- |
|  |  | Diffuse, moderate/strong | Diffuse, weak | Focal, moderate/strong | Focal, weak | No viable tumor cells | Negative | Total |
| D4D6 TMA | Diffuse, moderate/strong | 5 | 1 | 0 | 0 | 0 | 2 | 8 |
|  | Diffuse, weak | 3 | 7 | 1 | 1 | 0 | 7 | 19 |
|  | Focal, moderate/strong | 1 | 0 | 0 | 0 | 0 | 0 | 1 |
|  | Focal, weak | 2 | 3 | 0 | 9 | 0 | 12 | 26 |
|  | No viable tumor cells | 0 | 0 | 0 | 1 | 12 | 17 | 30 |
|  | Negative | 4 | 13 | 0 | 24 | 7 | 789 | 837 |
|  | Total | 15 | 24 | 1 | 35 | 19 | 827 | 921 |

## Table S5. Table including DNA NGS with CNV and hotspot mutations

| **Scoring group** | **Negative** | **Focal, weak** | **Focal, moderate/strong** | **Diffuse, weak** | **Diffuse, moderate/strong** | **No viable tumor cells** |
| --- | --- | --- | --- | --- | --- | --- |
| **D4D6 TMA**  **N (%)** | **837 (90.9)** | **26 (2.8)** | **1 (0.1)** | **19 (2.1)** | **8 (0.9)** | **30(3.3)** |
| Mean/median H-score (range) | 0 | 18.7/20 (5-40) | 35/35 (35) | 82,6/80 (60-130) | 221.3/220 (160-300) |  |
| FISH positive | 2/68 | 0/5 | 0/1 | 0/19 | 3/7 | 0/1 |
| RNA NGS  Number analyzed/total number in the group | 69/837 | 7/26 | 1/1 | 18/19 | 8/8 | 1/30 |
| --Fusions | ALK: 1  FGFR3: 1  MYB: 1  RET: 1  Failed: 2  Negative: 63 | Negative: 6  Failed: 1 | Negative: 1 | Met exon 14 skipping: 1  Failed: 1  Negative: 16 | ROS1: 3  Negative: 5 | Negative: 1 |
| DNA NGS  Number analyzed/total number in the group | 69/837 | 7/26 | 1/1 | 18/19 | 8/8 | 1/30 |
| --CNV | AKT2, FGFR1, CCND1, CCNE1, CDK6, FGF19 and FGF3: 1  Failed: 4  Negative: 61 | Negative: 7 | Negative: 1 | FGFR1, MDM2, MYC, PIK3CA, PDGFRA and KIT: 1  Negative: 14 | AKT2, AKT3, TERT, RICTOR and EGFR: 1  Negative: 4 | Negative: 1 |
| --Mutations | TP53: 31  EGFR: 9  KRAS: 6  PTCH1: 4  NFE2L2: 4  PTEN: 4  RAD50: 4  SETD2: 3  STK11: 3  PIK3CA: 3  ARID1A: 2  CREBBP: 2  FBXW7: 2  PIK3R1: 2 Failed: 3  Negative 15  NOTCH1, RB1, ERBB2, ESR1, MET, NOTCH3, CDKN2A and SLX4: 1 of each | TP53: 4  KRAS: 2  EGFR: 1  ERBB2: 1  STK11: 1  TSC1: 1  CDK4: 1  NOTCH3: 1  NRAS: 1  STAT3: 1 | KRAS: 1 | KRAS: 10  TP53: 6  SETD2: 2  Negative: 2  RB1: 4  ARID1A: 1  EGFR: 1  IDH1: 1  NOTCH1: 1 | TP53: 6  KRAS: 1  ARID1A: 1  BRCA2: 1  PTCH1: 1  SETD2: 1  Negative: 1  TSC2: 1  ATRX: 1 | TP53: 2  FANCD2: 1 |

| **Scoring group** | **Negative** | **Focal, weak** | **Focal, moderate/strong** | **Diffuse, weak** | **Diffuse, moderate/strong** | **No viable tumor cells** |  |
| --- | --- | --- | --- | --- | --- | --- | --- |
| **SP384 TMA**  **N (%)** | **827 (89.8)** | **35 (3.8)** | **1 (0.1)** | **24 (2.6)** | **15 (1.6)** | **19 (2.1)** |  |
| Mean/median H-score (range) | 0 | 25,3/30 (5-40) | 40/40 (40) | 100,4/100 (60-140) | 204/200 (120-300) |  | |
| FISH positive (Positive/total tested) | 2/56 | 0/7 | 0/1 | 0/21 | 3/15 | 0/1 | |
| RNA NGS  Number analyzed/total number in the group | 60/827 | 4/35 | 1/1 | 23/24 | 15/15 | 1/19 | |
| --Fusions | FGFR3: 1  MYB: 1  Failed: 2  Negative: 56 | RET: 1  Negative: 3 | Negative: 1 | ALK: 1  MET: 1  Failed:1  Negative: 20 | ROS1: 3  Failed: 1  Negative: 11 | Negative: 1 | |
| DNA NGS  Number analyzed/total number in the group | 60/827 | 4/35 | 1/1 | 23/24 | 15/15 | 1/19 | |
| --CNV | FGFR1: 2  PDGFRA: 2  AKT2, MYCL, PIK3CA, CCND1, CCNE1, CDK6, FGF19, KIT and FGF3: 1  Failed: 4  Negative: 49 | Negative: 4 | MYC: 1 | FGFR1: 22  MDM2: 1 | FGFR1: 12  AKT2, AKT3, TERT, RICTOR and EGFR: 1 | Negative: 1 | |
| --Mutations | TP53: 30  KRAS: 8  ARID1A: 4  NFE2L2: 4  PTCH1: 3  SETD2: 3  EGFR: 3  RB1: 3  RAD50: 3  NOTCH1: 2  FBXW7: 2  PIK3CA: 3  PTEN: 3  ERBB2: 1  TSC1: 1  CREBBP: 1  ESR1: 1  MET: 1  PIK3R1: 1  NRAS: 1  CDK4: 1  CDKN2A: 1  SLX4: 1  Failed: 3  Negative: 11 | TP53: 3  EGFR: 1  Negative: 1 | RB1: 1  NOTCH3: 1 | KRAS: 7  TP53: 6  EGFR: 6  STK11: 2  CREBBP: 1  PIK3R1:  RAD50: 1  NOTCH3: 1  STAT3: 1  Negative 5 | KRAS: 5  TP53: 5  PTCH1: 2  BRCA2: 1  SETD2: 1  EGFR: 1  IDH1: 1  ERBB2: 1  PTEN: 1  TSC2: 1  ATRX: 1  Negative: 1 | TP53: 1  FANCD2: 1 | |

## Table S6. IHC and stage, sex and smoking

|  | **Totally negative**  **D4D6/SP384** | **Focal, weak**  **D4D6/SP384** | **Focal, moderate/strong D4D6/SP384** | **Diffuse, weak**  **D4D6/SP384** | **Diffuse, moderate/strong**  **D4D6/SP384** | **No viable tumor cells**  **D4D6/SP384** |
| --- | --- | --- | --- | --- | --- | --- |
| **Median age** | 67.3/67.5 | 71.8/68.2 | 73.4/65.5 | 67.9/69.9 | 64.7/64.6 | 67.3/68.6 |
| **Female** | 402/401 | 14/17 | 1/0 | 10/10 | 4/11 | 16/8 |
| **Male** | 435/426 | 12/18 | 0/1 | 9/14 | 4/4 | 1411 |
| **Stage Ia** | 235/227 | 7/10 | 1/0 | 7/10 | 1/5 | 8/7 |
| **Ib** | 217/214 | 4/11 | 0/0 | 6/5 | 2/6 | 13/6 |
| **IIa** | 161/158 | 6/8 | 0/0 | 3/5 | 3/2 | 3/3 |
| **IIb** | 83/87 | 6/4 | 0/0 | 1/1 | 2/1 | 3/2 |
| **IIIa** | 126/126 | 3/2 | 0/1 | 1/2 | 0/1 | 3/1 |
| **IIIb** | 3/3 | 0/0 | 0/0 | 0/0 | 0/0 | 0/0 |
| **IV** | 10/10 | 0/0 | 0/0 | 1/1 | 0/0 | 0/0 |
| **Unknown** | 2/2 | 0/0 | 0/0 | 0/0 | 0/0 | 0/0 |
| **Never smoking** | 56/48 | 5/5 | 0 | 0/6 | 3/5 | 0/0 |
| **Former smoker** | 395/389 | 13/21 | 1/1 | 12/10 | 3/5 | 13/11 |
| **Smoker** | 386/390 | 8/9 | 0 | 7/8 | 2/5 | 17/8 |

## Table S7. TMA versus whole sections

|  |  | **D4D6 whole sections** | | | | |  |  |
| --- | --- | --- | --- | --- | --- | --- | --- | --- |
|  |  | Diffuse, moderate/strong | Diffuse, weak | Focal, moderate/strong | Focal, weak | Negative | N.A. | Total |
| **D4D6 TMA** | Diffuse, moderate/strong | 5 | 1 | 1 | 0 | 0 | 1 | 8 |
|  | Diffuse, weak | 4 | 8 | 0 | 5 | 2 | 0 | 19 |
|  | Focal, moderate/strong | 1 | 0 | 0 | 0 | 0 | 0 | 1 |
|  | Focal, weak | 0 | 2 | 0 | 2 | 1 | 21 | 26 |
|  | No viable tumor cells | 0 | 0 | 0 | 0 | 1 | 29 | 30 |
|  | Negative | 2 | 5 | 0 | 7 | 52 | 771 | 837 |
|  | Total | 12 | 16 | 1 | 14 | 56 | 822 | 921 |

|  |  | SP384 whole sections | | | | |  |  |
| --- | --- | --- | --- | --- | --- | --- | --- | --- |
|  |  | Diffuse, moderate/strong | Diffuse, weak | Focal, moderate/strong | Focal, weak | Negative | N.A. | Total |
| **SP384 TMA** | Diffuse, moderate/strong | 12 | 1 | 1 | 0 | 1 | 0 | 15 |
|  | Diffuse, weak | 6 | 12 | 0 | 1 | 2 | 3 | 24 |
|  | Focal, moderate/strong | 1 | 0 | 0 | 0 | 0 | 0 | 1 |
|  | Focal, weak | 0 | 3 | 1 | 1 | 0 | 30 | 35 |
|  | No viable tumor cells | 0 | 0 | 0 | 0 | 1 | 18 | 19 |
|  | Negative | 2 | 2 | 0 | 6 | 46 | 771 | 827 |
|  | Total | 21 | 18 | 2 | 8 | 50 | 822 | 921 |
